# Demand forecasting of cold-chain logistics of aquatic products in China under the background of the Covid-19 post-epidemic era

**DOI:** 10.1371/journal.pone.0287030

**Published:** 2023-11-30

**Authors:** Shuai Liu, Le Chang, Lin Wang

**Affiliations:** School of Marketing Management, Liaoning Technical University, Huludao, China; National Kaohsiung University of Science and Technology / Industrial University of Ho Chi Minh, TAIWAN

## Abstract

In the background of the post-epidemic era, the consumption demand and market scale of cold chain logistics in China are expanding, but there is still an obvious gap with developed countries. To complete the balance between the supply and demand for aquatic products and the rational allocation of logistics resources and promote the rapid development trend of aquatic product cold chain logistics, it is particularly important to forecast and analyze the demand for aquatic product cold chain logistics. This article selects six main factors that affect the demand for aquatic products in cold chain logistics, uses the traditional grey model and the grey-BP neural network model to simulate and predict the demand for aquatic products in cold chain logistics in China from 2012 to 2021, and compares and analyzes the simulation results. Generally speaking, the demand for aquatic products from Chinese residents is on the rise. In the simulation prediction process, the prediction error of the grey-BP neural network is reduced compared to the traditional grey model, and the processing ability of the nonlinear system is ideal. The results show that the grey-BP neural network model is an effective method to predict the demand for cold chain logistics of aquatic products. Finally, suggestions are made on the future development of aquatic cold chain logistics in the post-epidemic era from the economic, social, and environmental aspects, which provide valuable decision-making reference for the development of marine aquaculture enterprises and cold chain logistics industry.

## Introduction

On a global scale, the public health crisis caused by the Covid-19 epidemic, as an exogenous emergency, has had a broad and profound impact on the economic and social fields of various countries. Although the epidemic situation is improving, its diffusion and derivative effects continue [1]. The cold chain logistics industry is widespread in all walks of life, which is an important industry to consolidate the achievements of poverty alleviation and difficulty management, effectively linking rural revitalization, and promoting the improvement of consumption. The release of the "^14th^Five-Year Plan for the Development of Cold Chain Logistics" puts forward clearer requirements for the development of cold chain logistics in the post-epidemic era, and the cold chain logistics industry will become an important force in building a new development pattern of double circulation. The spoilage rate of aquatic products in China is about twice that in developed countries. From the perspective of the cold chain transportation rate of fresh products, the cold chain transportation rate of aquatic products in China is 69% and the average cold chain transportation rate of aquatic products in developed countries is as high as 95%. Compared to developed countries, China lacks cold chain hardware facilities and unevenly distributes equipment. The cold chain infrastructure is mainly concentrated in coastal areas and developed first-tier cities; the central and western regions, which carry out most of the wholesale transactions of fresh agricultural products in China, are short of cold chain resources, and their development lags. In the context of the post-epidemic era, new changes and characteristics have emerged in the global aquatic product supply chain, and China’s fishery has been gradually impacted by the general situation. The development of China’s cold chain logistics is faced with enormous challenges all the time. In summary, forecasting and analyzing the demand for cold chain logistics in China can ensure the safety of aquatic food and the efficient operation of cold chain logistics in the post-epidemic era. To complete the balance between supply and demand [2] of aquatic products and rational allocation of logistics resources and to reasonably promote the rapid development trend of aquatic cold chain logistics [3, 4], it is particularly important to forecast the demand for aquatic cold chain logistics in China.

In 1894, Barrier and Ruddich first proposed the concept of the cold chain [5]. In the 1940s, the cold chain received enough attention and developed rapidly. Since then, many scholars have studied cold chain logistics from different angles and have obtained rich research results. The Cold Chain Association (CCA) was established in the United States in February 2003, which is committed to the research of perishable goods and temperature-sensitive products, and the formulation of relevant standards for cold chain transport [6]. In the following year, the Cold Chain Quality Index (CCQI) was released to test the reliability, quality, and proficiency of enterprises related to cold chain logistics, laying the foundation for perishable goods supply chain certification. China’s cold-chain logistics began in the 1960s, and the promulgation of the Food Health Law of the People’s Republic of China in 1982 promoted the development of food cold-chain logistics. With the development of economic construction and the continuous improvement of the living standards of people, the requirements for food safety are getting higher and higher [7]. As an important part of the food safety system, cold chain logistics has developed rapidly. From the point of view of the whole cold chain system, the cold chain has not yet formed a system, be it due to the demand for consumption of Chinese economic development, or compared with the developed countries, the gap between the two is very significant [5].

In the field of academic research, demand forecasting research focuses mainly on the analysis of the current demand situation for cold chain logistics, countermeasures, and research methods for agricultural products. Most scholars focus on the analysis of the current situation of agricultural products’ cold-chain logistics demand in various provinces and cities, countermeasures and suggestions, and other qualitative research parts. For example, Dongfang et al. [8] presented countermeasures and suggestions for the development of cold chain logistics in community fresh retail business based on the dual perspectives of new consumption demand and service improvement, and Zhang et al. [9] identified the weak links of cold chain logistics of fresh agricultural products in China and presented countermeasures. Leng et al. [10] studied the development status and problems of cold chain logistics in Hubei province based on the new normal. Many scholars have emphasized the importance of the development of cold chain logistics for China’s economy and the problems and countermeasures in the process of development. It can be seen that the prediction accuracy of the grey prediction model and the BP neural network model has been recognized by many scholars in the field of logistics demand prediction. The grey prediction model and the BP neural network model are often combined in the field of prediction. For example, Chen X [11] conducted an early warning study on the financial risk of H Company based on the grey BP neural network model; Zhang X et al. [12] used the model to forecast the throughput of hazardous chemicals in the Zhoushan port of Ningbo; Lu J et al. [13] used the grey BP neural network to forecast the cold chain logistics demand for aquatic products in Dalian, and pointed out that compared to other prediction models, The combined model has higher prediction accuracy in the field of prediction of cold chain logistics demand for aquatic products.

In terms of research methods, researchers use the BP neural network [14], the RBF (Radial Basis Function) neural network [15], the support vector regression [16], machine learning [17, 18], the optimal combination forecasting model [19, 20], and other methods to predict the development and change laws of things in different fields and show good prediction results. Foreign scholars compare the quantitative research methods of this research, for example, Amir Shaban [21] compares the prediction conditions of four models: the moving average method, the weighted moving average method, the exponential smoothing method, and the grey prediction method under the condition of supply chain interruption and stability. The simulation shows that the grey prediction method is extremely stable. Benkachcha et al. [22] compared the forecast accuracy of multiple linear regression and artificial neural network forecasting models for supply chain demand, and finally concluded that artificial neural network forecasting models can forecast demand more effectively and prepare for it. Can Eksoz et al. [23] summarized the grey model, the neural network model, and the combination forecasting model, and comprehensively considered the factors affecting demand, making the short-term forecast of the demand for cold chain logistics more scientific and accurate.

**Fig 1:**
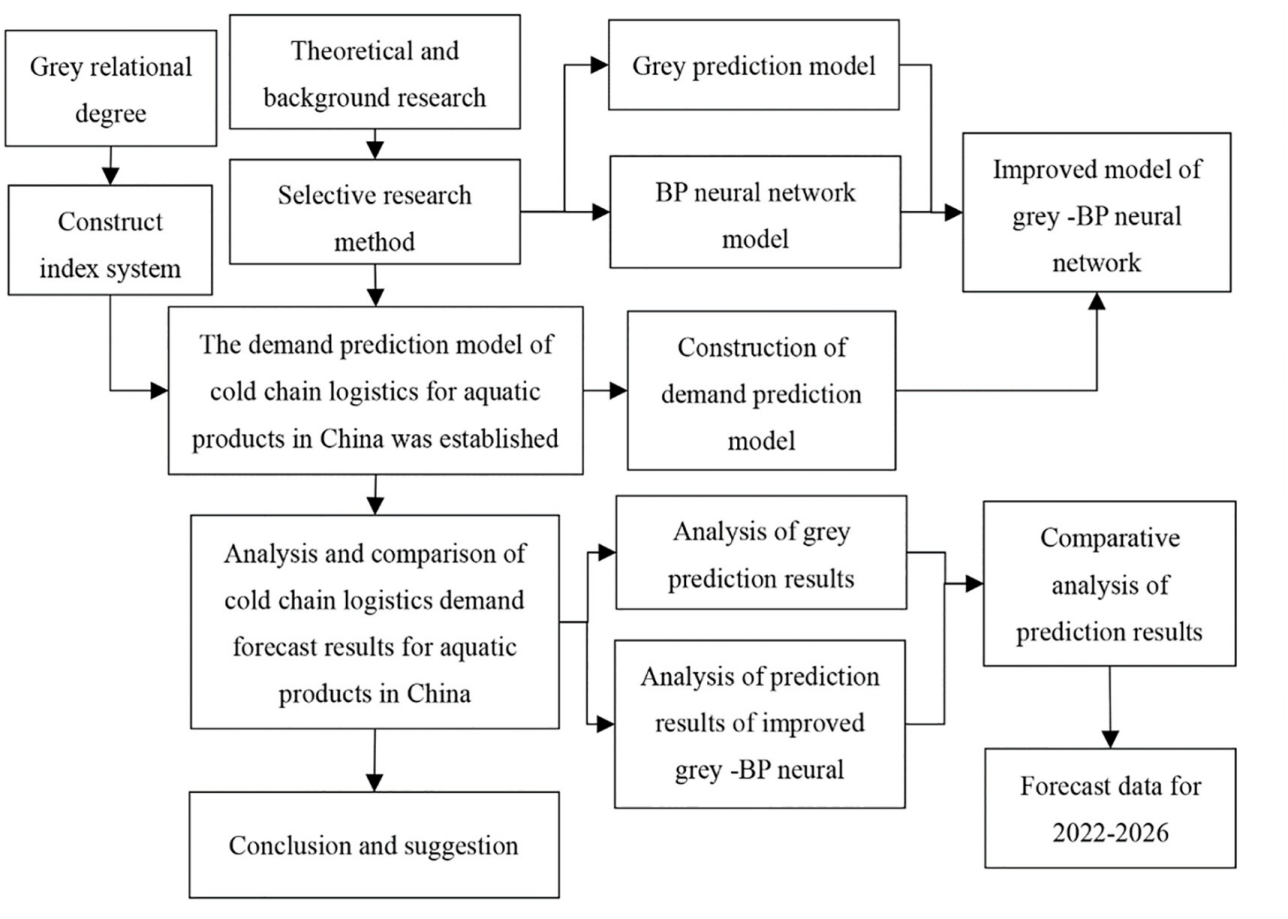
The flowchart of the study process.

In current related research, cold chain logistics lacks relevant statistical data, and there is no perfect measurement index system, so there are still many difficulties in forecasting the demand for cold chain logistics. Looking at the research results of various scholars, the grey forecasting model and the BP neural network model are the most accurate forecasting methods in cold chain logistics demand forecasting. Therefore, this paper constructs an index system from two aspects of economic factors and supply factors, adopts the forecasting method of combining grey system forecasting with BP neural network forecasting, and improves it to meet the needs of aquatic product logistics demand forecasting. The flowchart of the study process is shown in Fig 1.

## Research methods

Logistics demand forecasting is based on logistics data related to market demand and demand conditions and scientifically predicts future logistics demand. According to the simulation results, companies can accurately understand the trend of market demand and adopt the corresponding measures to contribute to the interests of companies. Logistics demand can be predicted using empirical forecasting methods and mathematical model methods. In this paper, the mathematical model method is used to study the demand for cold chain logistics of aquatic products in China. The specific mathematical model is as Eq (1).


$$Y=f\left(x_{1},x_{2},\ldots ,x_{n}\right)$$ (1)

Assuming logistic demand as Y, x1-xn as factors affecting logistics demand, economic factors, social factors, technical factors, and environmental factors are the main factors. Subsequently, the grey model is established for the cold chain logistics demand system for aquatic products in China, and the dynamic model of GM (1, 1) is adopted in this article. If the original data sequence of logistics demand is set to *x*^(1)^, the accumulated new data sequence is *x*^(1)^, *α* and *u* are the parameters to be estimated, Then *x*^(1)^ satisfies the first-order differential equation as Eqs (2 and 3).


$$\frac{dx^{\left(1\right)}}{dt}+\alpha x^{\left(1\right)}=u$$ (2)

$${\hat{X}}^{\left(1\right)}\left(i+1\right)=\left(X^{\left(1\right)}-\frac{u}{\alpha }\right)e^{-\alpha i}+\frac{u}{\alpha }$$ (3)

The least squares fitting method is used to solve the parameters *α* and *u* to be estimated as Eqs (4, 5 and 6).

A^=αn=BTB-1BTYN (4)

Where

$$B=\left[\begin{array}{cc} -\frac{1}{2}[x^{\left(1\right)}\left(1\right)+x^{\left(1\right)}\left(2\right) & 1\\ -\frac{1}{2}[x^{\left(1\right)}\left(1\right)+x^{\left(1\right)}\left(3\right) & 1\\ \ldots & \ldots \\ -\frac{1}{2}[x^{\left(1\right)}\left(m-1\right)+x^{\left(1\right)}\left(m\right) & 1 \end{array}\right]$$ (5)

$$YN\;=\left(x\left(0\right)\left(2\right),\;x\left(0\right)\left(3\right),\ldots ,x\left(0\right)\left(m\right)\right)T$$ (6)

After *α* and *u* are obtained, the following grey prediction model can be obtained by entering the values into the first-order differential equation shown above as Eq (7).


$${\hat{X}}^{\left(1\right)}\;\left(t\;+\;1\right)=\left(x{\;}^{\left(0\right)}\left(1\right)-\frac{u\;}{\alpha }\right)\;e-\alpha t\;+\;\frac{u\;}{\alpha }$$ (7)

**Fig 2:**
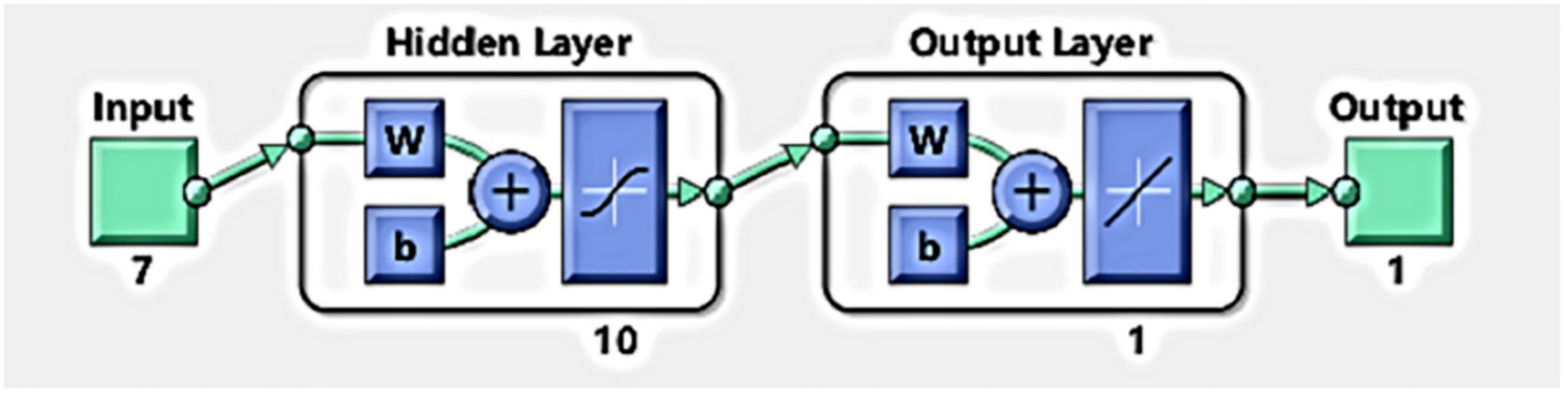
Schematic diagram of the neural network structure.

The BP neural network (Fig 2) [24] is preferred by many scholars due to its strong operability and simple structure. The Fig 2 is derived from the matlab2021a neural network toolbox. It has a strong nonlinear input-output relationship simulation ability, strong nonlinear approximation ability, generalization ability, and ease of use, and can well simulate logistics market systems, but the convergence speed is slow. The activation function in the BP neural network is as Eq (8).


$$f\left(x\right)=\frac{1}{1+e^{-2n}}-1$$ (8)

**Fig 3:**
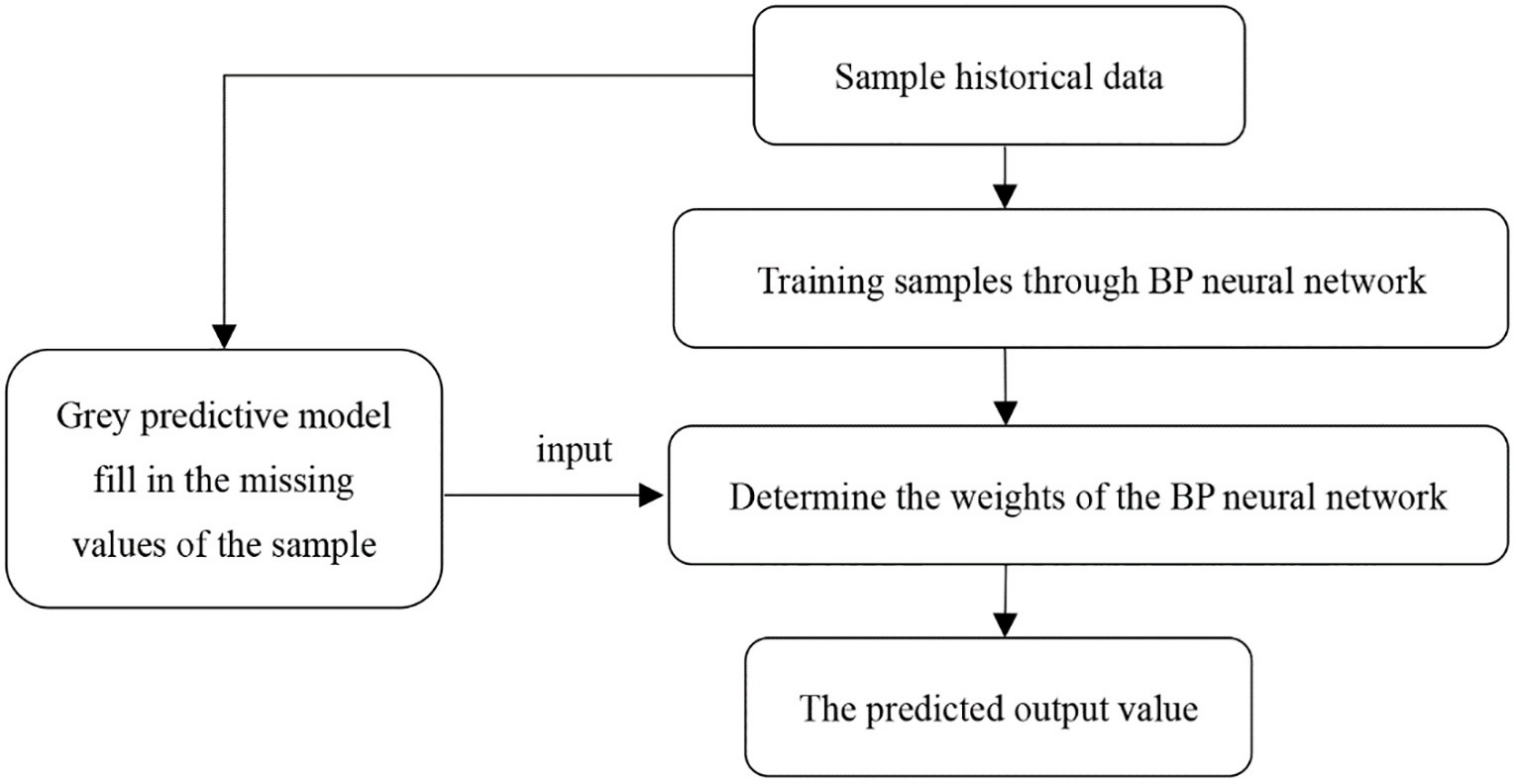
The improved model of grey-BP neural network.

Although the grey prediction model is simple to calculate and has a remarkable simulation effect, it is deficient in complex nonlinear functions. BP neural network can neutralize the grey prediction model, is difficult to deal with non-linear problems, and easy to adapt to strong, the advantages and disadvantages of the two models complement each other, set their strengths, and can achieve further improvement of prediction accuracy. Combining the grey forecasting model with the BP neural network [13] can avoid errors and defects caused by forecasting by a single model; at the same time, the convergence rate is increased, the improved model is more suitable for forecasting cold chain demand. The improved grey-BP neural network model constructed in this paper is shown in Fig 3. The historical data sample is input into the grey prediction model to obtain the preliminary grey predicted value and fill the missing value of the sample. At the same time, the historical data sample is carried out BP neural network training, combined with the index data studied by many scholars to determine the weight of the BP neural network, the data obtained from the grey prediction model is input into the BP neural network to determine the weight, and finally the predicted output value is obtained.

## Empirical research

### Analysis and selection of influencing factors

Based on research from many scholars, this paper establishes the forecast index system of the cold chain demand for aquatic products. Relevant data comes from the official website of the National Bureau of Statistics, the China Fishery Yearbook 2012–2021, the China Statistical Yearbook, the China Cold Chain Logistics Development Report, etc. The original experimental data are sorted out by direct citation or indirect calculation. The grey prediction model is used to fill in the missing data. Among them, because there are no official demand data, this paper refers to the literature on the prediction of cold chain logistics demand for Hainan-Free Trade Port agricultural products based on the grey Markov chain of Li et al. [25], and chooses the production of aquatic products as a substitute value for the cold chain logistics demand. Most scholars use the total amount of each index to study, so the prediction of the population base will have a noticeable impact on the prediction results. In this paper, per capita data are used in data selection, which avoids the impact of population based on prediction results and greatly improves the accuracy of demand prediction for aquatic product cold chain logistics.

#### Economic factor

1. Economic development level: On the backdrop of rapid economic development, China has a demand for various aquatic products and related products, driving the demand for cold chain transportation to increase and enhancing the demand for cold chain logistics. Regional GDP can effectively express the level of economic development within the region, so the level of economic development in this article is expressed by China’s GDP.
2. Industrial structure: Logistics is the tertiary industry. The degree of development of the tertiary industry in the region is the decisive factor for the requirements of the logistics service, which promotes the rapid development of the logistics industry, and the demand for logistics is determined by the industrial structure in China. Because cold chain logistics does not determine the numerical proportion of industrial structure, the industrial structure in this paper is expressed by the proportion of the output value of China’s tertiary industry to GDP.
3. Per capita disposable income: With the progress of society and the rapid development of the economy, the consumption structure and the concept of consumption of Chinese residents have slowly changed in the process of social development [26]. People no longer seek delicious food with high oil and high fat, and their eating habits have gradually changed, preferring healthy and low-fat foods. Aquatic products have long been the preferred food for people due to their low-fat and high-protein characteristics. In recent years, people’s pursuit of health has increased day by day, which increases people’s demand for aquatic products. In the backdrop of the post-epidemic era, consumers are more concerned about the safety of aquatic products and have stricter requirements for cold chain logistics transportation. Therefore, this paper also brings disposable income per capita into the index system.

#### Supply factors

1. The production of aquatic products: The production of aquatic products is a direct factor affecting the supply of aquatic products, and the supply further affects people’s demand for aquatic products. In the process of increasing people’s demand, cold chain logistics, a derivative demand, should provide supporting supply and services. Therefore, the total production of aquatic products plays a very important role in this.
2. Consumer price index of aquatic products: Urban consumer price index can reflect the relative quantity and fluctuation degree of residents’ prices in a certain period, and can also observe the impact of price fluctuation on residents’ consumer goods and services on residents’ lives. The consumer price index of aquatic products can represent the impact of the retail price of aquatic products on the lives of citizens. The increase and decrease of the index can directly express the purchasing power of residents and indirectly affect the increase or decrease in consumers’ demand for aquatic products.

#### Technical factors

The fishing time for aquatic products is fixed and they are unloaded centrally during the non-fishing season every year. The number and refrigeration capacity of cold storage are related to the quality of aquatic products. Cold storage is the core component of cold chain logistics. According to data released by the International Cold Storage Warehouse Association (IARW) in 2019, the per capita storage capacity in the United States reached 0.49 cubic meters per person in 2018, 0.32 cubic meters per person in Japan, and only 0.14 cubic meters per person in China by 2021. According to the statistics of the 2021 edition of the Intercooling Alliance National Cold Chain Logistics Enterprise Distribution Map, from 2017 to 2021, China’s cold storage capacity increased from 36.09 million tons to 52.24 million tons, with a compound annual growth rate of 9.7%, maintaining a stable growth rate. Therefore, in this article, the per capita cold storage capacity is included in the cold chain logistics demand forecasting index system of aquatic products. See Table 1 for specific data [27].

**Table 1:** Forecast index system of cold chain logistics demand for aquatic products.

Year	Per capita GDP (yuan)	Per capita disposable income (yuan)	Residents’ consumption level (yuan)	The proportion of tertiary industry in GDP (%)	The consumer price index of aquatic products (last year = 100)	Per capita cold storage capacity (m^3^/person)	Per capita demand (kg/person)
**2012**	39771	16510	14074	45.5	108	0.039	40.63
**2013**	43497	18311	15586	46.9	104.2	0.044	42.01
**2014**	46912	20167	17220	48.3	104.4	0.060	43.6
**2015**	49922	21966	18857	50.8	101.8	0.068	44.9
**2016**	53783	23821	20801	52.4	104.6	0.075	45.82
**2017**	59592	25974	22968	52.7	104.4	0.085	46.03
**2018**	65534	28228	25245	53.3	102.3	0.093	45.95
**2019**	70078	30733	27504	54.3	100.3	0.107	45.96
**2020**	71828	32189	27439	54.5	103	0.125	46.38
**2021**	81370	35128	31013	53.5	109.4	0.139	47.36

## Results and analysis

### Data preprocessing

Because of the different dimensions among the indicators in this paper, the data are incomparable, so it is necessary to carry out dimensionless processing on the data, and eliminate the dimensional influence of the original variables through data transformation for subsequent analysis. In this paper, the normalization processing method is used to eliminate the influence of dimensions. The specific formula is Eq (9).


$$x_{i}=\frac{x-x_{min}}{x_{max}-x_{min}}$$ (9)

### Grey relational grade analysis

To ensure the precision of the forecast results, this article performs a correlation analysis of the data in the index system and uses the grey correlation method to perform a correlation analysis of the demand for cold chain logistics of aquatic products in China and six influencing factors. The value of the degree of correlation is between 0 and 1, and the larger the value, the stronger the correlation between it and the demand for cold chain logistics of aquatic products, which means the higher the evaluation. It can be seen from Table 2 that the degree of correlation of the six indexes in this paper is greater than 0.9, and the degree of correlation is strong, so the selection of the index is suitable for the prediction of the demand for cold chain logistics of aquatic products.

**Table 2:** Results of the grey relational degree.

Evaluation items	Degree of relevance	Rank
**Per capita GDP (100 million yuan)**	0.9321	6
**Per capita disposable income (yuan)**	0.9334	2
**Residents’ consumption level (yuan)**	0.9329	3
**The tertiary sector as a percentage of GDP**	0.9351	1
**The consumer price index of aquatic products (last year = 100)**	0.9326	4
**Per capita cold storage capacity (cubic meters/person)**	0.9323	5

### Grey prediction model

The grey forecasting model method is used to analyze the relevant data for China from 2012 to 2021, and the forecast value of per capita demand of the grey forecasting model is obtained. Specific results and analysis are as follows.

**Fig 4:**
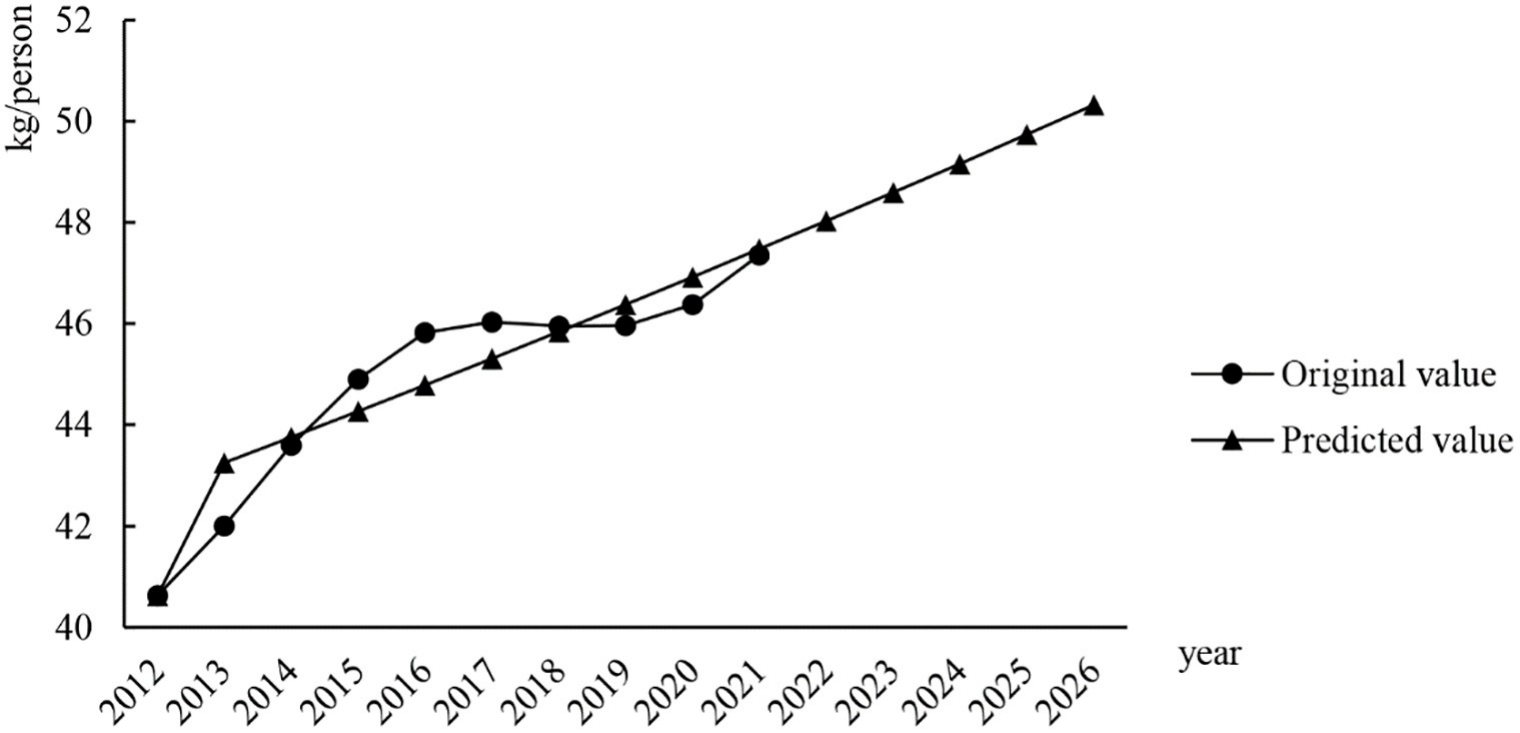
Grey predictive model fitting and prediction.

The grey prediction model of the demand for cold chain logistics for aquatic products in China is shown in Table 3. From the table, we can observe the coefficient of development of model *a*, the grey action *b*, the posterior difference ratio *c*, and the small error probability *p*; The posterior difference ratio *c* is 0.099 < = 0.35, which means that the accuracy level of the model is very good. Furthermore, the small error probability *p* value is p = 1.000(≥1.0), which means that the accuracy of the model is very good. The grade ratio test values are all within the standard range [0.834, 1.199], which means that these data are suitable for model construction. The grey prediction model test table is shown in Table 4. From Table 4, it can be seen that the maximum relative error value of the model is 0.030 < 0.1, which means that the fit effect of the model meets the higher requirements. For the deviation value of the grade ratio, the value is less than 0.2, which means that it meets the requirements, and if it is less than 0.1, it means that it meets the higher requirements; The maximum relative error value of the model is 0.025 < 0.1, which means that the fitting effect of the model meets the higher requirements. The results of the per capita demand data for 2022 to 2026 predicted by the grey prediction model of the cold chain logistics demand of aquatic products of China are shown in Table 5, and the line chart of the original data fitting and prediction data is shown in Fig 4. From the chart, it can be found that the forecast data of China’s aquatic products cold chain logistics demand is consistent with the trend of the original data, but there is still a big deviation in the degree of adjustment between the specific prediction data and the actual value, which can only predict the overall trend of the data, and the reflection of a small fluctuation of the data is insufficient, so it is necessary to further explore a more accurate prediction method model.

**Table 3:** Model construction results.

Development factor *a*	Grey action amount *b*	Posterior difference ratio *c* value	Small error probability *p* value
-0.0117	42.5255	0.0988	1.000

**Table 4:** GM (1, 1) model checking table.

Ordinal	Original value	Predicted value	Residuals	Relative error	Grade deviation
**1**	40.630	40.630	0.000	0.000%	-
**2**	42.010	43.250	-1.240	2.953%	0.022
**3**	43.600	43.757	-0.157	0.361%	0.025
**4**	44.900	44.270	0.630	1.403%	0.018
**5**	45.820	44.789	1.031	2.250%	0.009
**6**	46.030	45.314	0.716	1.556%	-0.007
**7**	45.950	45.845	0.105	0.229%	-0.013
**8**	45.960	46.382	-0.422	0.919%	-0.011
**9**	46.380	46.926	-0.546	1.177%	-0.003
**10**	47.360	47.476	-0.116	0.244%	0.009

**Table 5:** Forecast data from 2022 to 2026.

Year	2022	2023	2024	2025	2026
**Predicted value**	48.032	48.595	49.164	49.741	50.324

### Improved model of grey-BP neural network

Based on the weak ability of the grey prediction model to reflect a small fluctuation of data, this paper uses the BP neural network model to integrate and improve it. Li et al. [28] use the improved grey neural network to predict the demand for coal logistics. The prediction results show that the model has high prediction accuracy and is feasible for complex nonlinear systems. Eksoz [29] predicts the short-term demand for cold chain rail logistics in a certain area using the grey model, the neural network model, and their combination model. The research shows that the combination forecasting model has a better forecasting effect than the single forecasting model. Yan [24] applied the combined model to logistics demand forecasting in Hunan province in research of logistics demand forecasting based on a grey neural network model. The experimental results show that the improved model improves the accuracy of logistics demand forecasting, gives full play to the advantages of two single models, overcomes the shortcomings of a single model, and provides an effective method for logistics optimization management. From the above research, we can find that the improved grey-BP neural network model is feasible to apply to complex nonlinear systems. Compared with a single prediction model, it has a better prediction effect and can give full play to the advantages of the model, which can make up for the lack of weak ability to reflect small fluctuations of data caused by using a single model.

**Fig 5:**
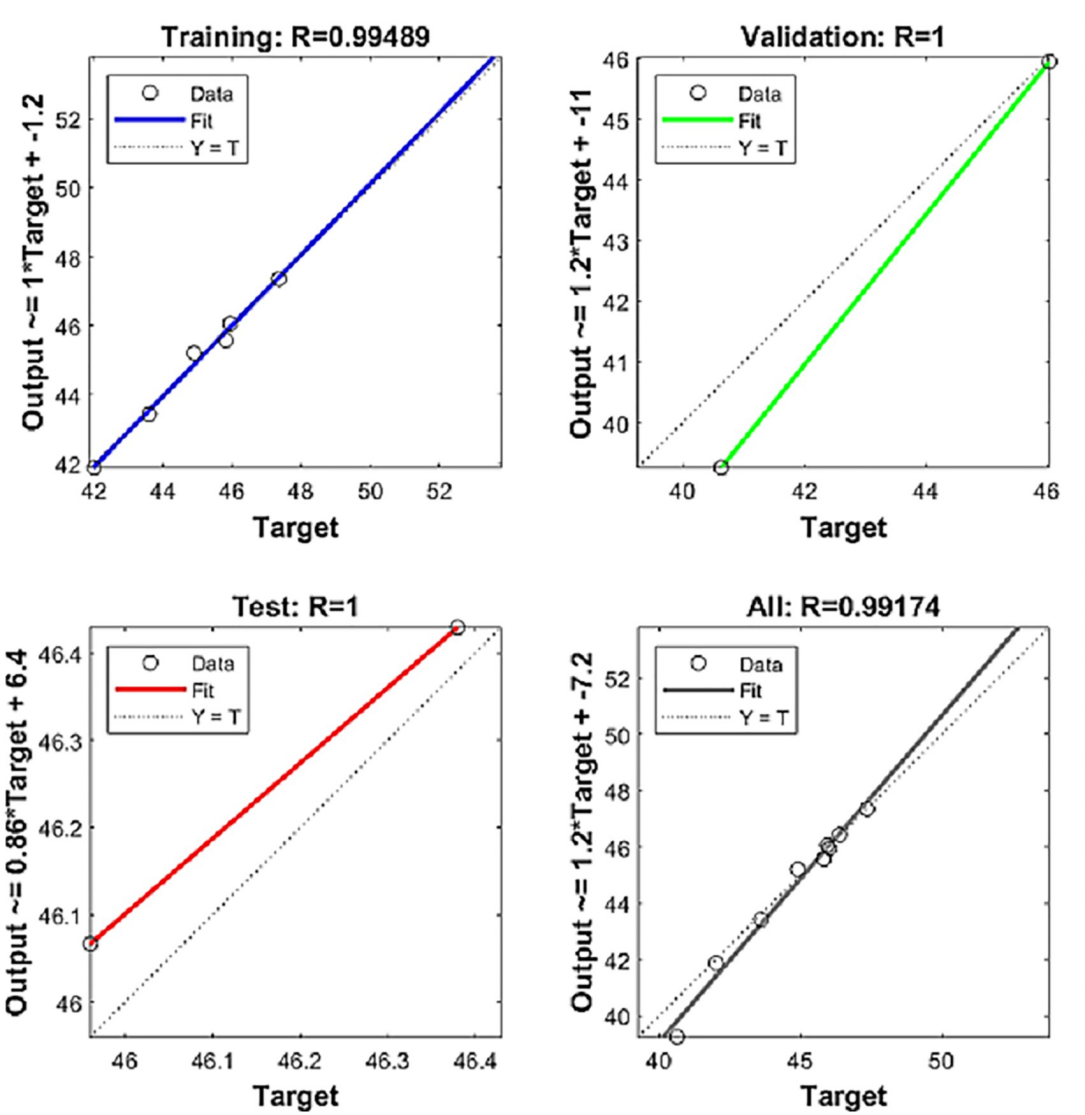
Fitting diagram of the neural network toolbox test model.

Based on the data obtained from the grey prediction model above, combined with the index data studied by many scholars, the BP neural network model is input with determined weights, and the test results shown in Table 6 are obtained using the neural network toolbox [24] provided by Matlab2021a. It can be seen from the test results that the MSE (mean-square error) value of the improved grey-BP neural network model for the cold chain logistics demand of aquatic products in China is low as a whole, and the R value is all 1, showing that the correlation degree between the model indicators is good. The degree of adjustment of the training set reaches 0.94886, which shows that the accuracy of the model is high. The specific results of the adjustment are shown in Fig 5.

**Table 6:** Test results of neural network toolbox.

	Samples	MSE	R
**Training**	11	3.53986e-1	9.94886e-1
**Validation**	2	9.46434e-0	9.99999e-1
**Testing**	2	6.91222e-0	9.99999e-1

**Fig 6:**
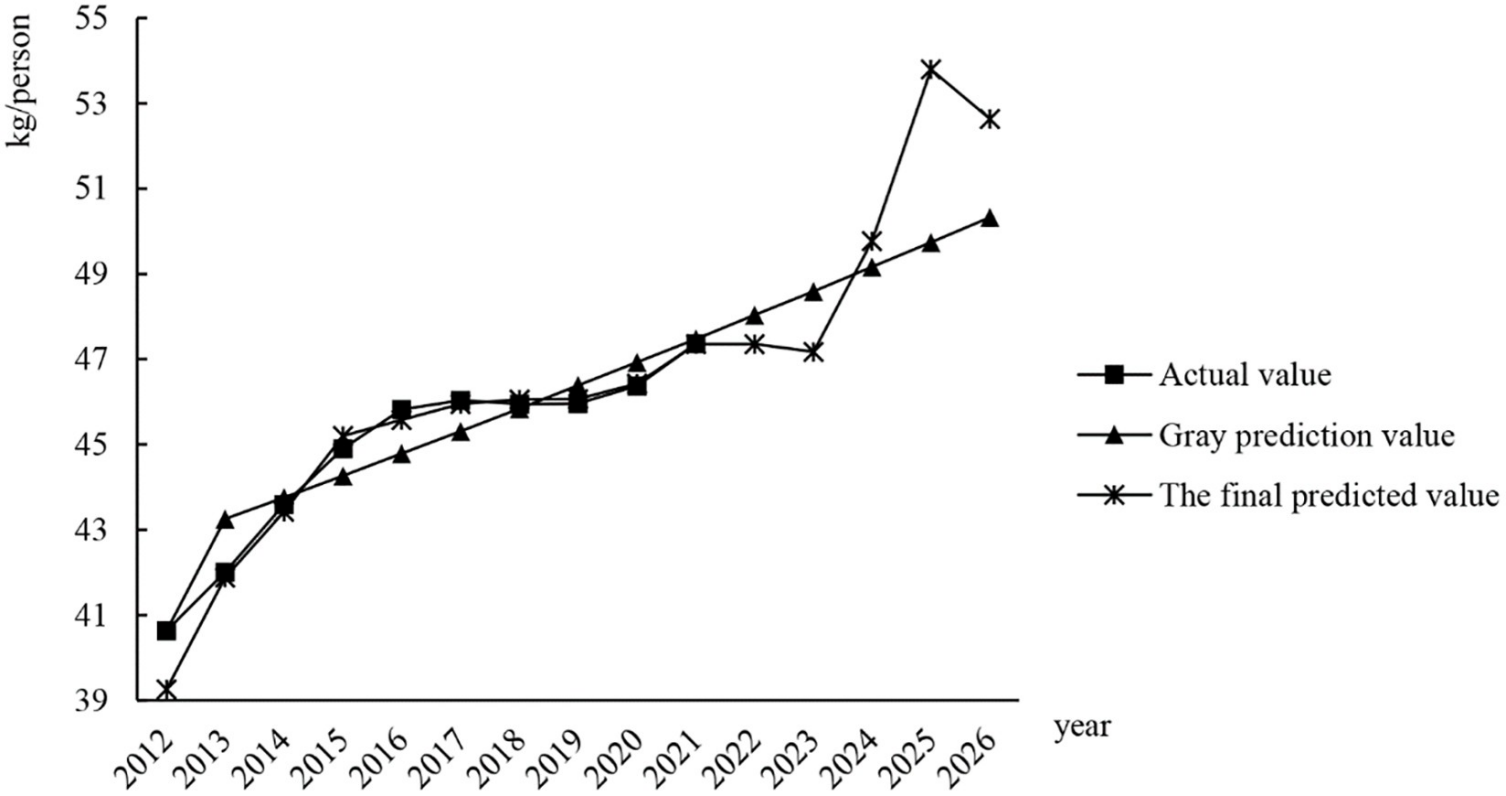
Model-fitting comparison diagram.

The comparison chart of the degree of adjustment of the demand for aquatic products and the prediction results of the model in China over the years is shown in Fig 6. It can be seen from the figure that the error between the final predicted value of the grey-BP neural network model and the actual value over the years is small, which can not only accurately fit the overall trend of the data, but also perfectly reflect the small fluctuation of the data. Although there are still some errors in individual data, the fitting result is much higher than the data accuracy of the traditional grey prediction model, and the overall trend and small fluctuation of the data are consistent with the actual value. Therefore, the model constructed in this paper is of great significance in predicting the cold chain demand for aquatic products in the future.

Using the previous model method, this article predicts the per capita demand for cold chain logistics of aquatic products in China from 2022 to 2026, and the specific data are shown in Table 7. Then, according to the fluctuation trend of the prices of aquatic products and the forecast of the per capita demand for cold chain logistics, the value of fishery production in China is predicted in the next five years. The specific forecast results are shown in Table 8.

**Table 7:** Per capita demand for cold chain logistics of aquatic products in China from 2022 to 2026.

Year	2022	2023	2024	2025	2026
**Per capita demand (kg/person)**	47.35	47.18	49.78	53.79	52.63

**Table 8:** The value of the fishery production in China from 2022 to 2026.

Year	2022	2023	2024	2025	2026
**China’s fishery production value (100 million yuan)**	14282.49	14130.96	14643.14	14424.73	14439.09

## Discussion

The predicted value of China’s cold chain logistics demand in 2022 is 47.35 kg per person, which is slightly different from the data in 2021, indicating that there is not much fluctuation. By 2023, the predicted value will show a downward trend. The predicted value of China’s fishery output value in 2022 is 1,428.249 billion yuan, which is approximately 22.478 billion yuan lower than that of China’s fishery output value of 1,450.727 billion yuan in 2021. The trend of China’s value of fishery production over the next five years is the same as that of per capita demand. Compared with the results obtained by the traditional grey forecasting model, the improved grey-BP neural network model used in this paper can greatly improve the forecast accuracy of cold chain logistics demand of aquatic products in China.

From a macro point of view, the output value of China’s fishery decreased by 22.478 billion in 2022 compared with that in 2021, but the demand hardly changed. The reason may be the fluctuation in the price of aquatic products caused by the COVID-19 epidemic. However, with the orderly and steady introduction of cold chain-related policies and control measures in China, these crisis problems have been successfully lifted or successively lifted in real life. After the full-scale outbreak of the COVID-19 epidemic in 2020, the government issued as many as seven cold chain policies related to the epidemic, which not only effectively guaranteed the steady operation of the cold chain logistics industry in the post-epidemic era, but also effectively reduced consumers’ doubts. Key policies include "Technical Guidelines for Prevention and Control of Cold Chain Food Production and Operation in SARS-COV-2" and "Technical Guidelines for Prevention and Control and Disinfection of Cold Chain Food Production and Operation in SARS-COV-2". To prevent cold chain agents and their stakeholders from infecting Novel Coronavirus as the main line, various measures are taken to strengthen the disinfection of cold chain food packaging in multiple dimensions. And in the "Implementation Opinions on Further Reducing Logistics Costs", 24 specific measures in six aspects are put forward to implement the "green channel" policy for the transportation of perishable agricultural products and reduce the consumption cost of perishable agricultural products under the background of human life. Optimize logistics vehicle management, fully guarantee timely and efficient product distribution, and ensure stable operation of the cold chain logistics system.

The main reasons for the decline in the forecast value in 2023 may be the following two points.

First, due to the fishing ban policy implemented in recent years to protect natural resources, the fish culture area and fishing production have been affected, and the epidemic situation has led to strict control of the import of aquatic products, which has greatly reduced the aquatic products and imported aquatic products in China. Therefore, the price of aquatic products continues to increase, leading to a continuous decline in the demand for aquatic products by our people.

Second, affected by the news of nuclear wastewater discharged from Japan. On 3 August 2022, local time, Tokyo Electric Power Company of Japan announced that the nuclear-polluted water discharge facility project of the Fukushima Daiichi Nuclear Power Plant will officially start on 4 August or begin discharge in the summer of 2023. The German Marine Research Institute conducted a study on the Fukushima current, and the results pointed out that radioactive materials may spread to most parts of the Pacific Ocean within three months, and radioactive materials will be detected in global waters ten years later, which will pose an incalculable threat to the inventory of marine life and the life and health of global citizens. Especially, it will have a great impact on Chinese fishery near Japan, and the polluted water quality will seriously affect the quality of seafood and the health of consumers. This series of reports will have a series of impacts on consumers’ psychology, and residents’ consumption structure will change unconsciously. In consumption, they avoid buying aquatic products and choose substitutes for meat, beans, milk, and other aquatic products to substitutes to absorb the required nutrients. Due to the influence of public opinion on consumers’ psychology, the consumption structure of residents has changed, resulting in a decline in the demand for aquatic products.

In 2024, the predicted value of China’s value of fishery production and the per capita demand for cold chain logistics both increased greatly, which should be related to national macro control and the slow recovery of economic and social order in the post-epidemic era [30]. At the same time, after the nuclear wastewater is discharged into the waters outside Fukushima, the polluted water source will not reach the Chinese waters for the first time. According to the North Pacific Ocean Current Map, nuclear waste water will drift northwest with the Japanese warm current, first reach Canadian waters with the North Atlantic warm current, then spread to the west coast waters of the United States driven by the California current, and finally reach Taiwan waters of China and further spread to East China Sea waters via the North Equatorial Warm Current. Nuclear wastewater needs to circulate in the North Pacific Ocean to reach China’s waters, and the whole process takes about 200 days. At the same time, China’s inland rivers inject water resources along the mainland coast, and the strong pressure difference will form a barrier and reduce the impact on offshore waters. It may be this reason that leads to the decline of China’s value of fish production after 2025.

## Conclusions and recommendations

In this article, the grey forecasting model combined with the BP neural network model is used to build a cold chain logistics aquatic product demand forecasting model, collate the relevant literature, determine the indicators required by the model, and combine it with the relevant statistical data from 2012 to 2021, using Matlab2021a software to test the improved BP neural network, grey forecasting model. It has high precision and ensures a scientific and reasonable forecast of the demand for aquatic products in China’s cold chain logistics. Through the above research, the following conclusions are drawn.

1. In the forecasting process, the overall variables are greatly influenced by the population base and the influence of population changes on the output of aquatic products over the years cannot be ruled out. The precision of the forecasting results in this paper is higher than that of Yan [31] who did not use the data of the variables per capita in the forecast of logistics demand. Therefore, the selection of per capita variables in the index selection can avoid the impact of population based on the prediction results to the greatest extent, and greatly improve the accuracy of the demand prediction of aquatic products cold chain logistics.
2. The traditional grey model needs fewer samples and is easy to use. However, in simulation prediction, irregular changes will have a great impact on the accuracy of simulation prediction. For samples with obvious trend fluctuation, the error is relatively large. The research results of this paper are consistent with those of Li et al. [32]. The aquatic product cold chain logistics system is complex and affected by many factors, and the relationship between the systems is nonlinear in most cases. However, they only use a single model for comparison and do not combine multiple models for comparison and prediction. The neural network has great advantages in dealing with complex non-linear systems. Combining the traditional grey model with the BP neural network can predict the demand for cold chain logistics more accurately.
3. In the forecasting process for the demand for aquatic products in cold chain logistics, the result of the relative error prediction of the grey-BP neural network is smaller and more accurate than that of a traditional single prediction. Xu et al. [33] have shown that the grey-BP neural network combines the forecasting of GM (1, 1) with the BP neural network, which can effectively deal with the forecasting of the cold chain logistics of aquatic products affected by strong complexity, nonlinearity, and multiple factors, to achieve the effect of a highly suited nonlinear relationship. The grey forecasting model and the BP neural network model are the most effective forecasting methods for aquatic products cold chain logistics, which can accurately analyze the nonlinear relationship, especially in complex systems, provide more valuable background information for decision-makers, improve the demand planning of cold chain logistics, and contribute to the sustainable development in the cold chain logistics industry. However, the selection of research objects of the grey-BP neural network is not suitable for the analysis of single factor and simple linear relationships, which may show complex trends, slow convergence speed, poor reliability, and other shortcomings. It is very important to select different models according to the characteristics of different data. In the future, the research field of grey-BP neural network will be extended to fresh agricultural products and the cold chain drug industry.

The comparison between the results of this paper and those of previous studies is shown in the Table 9.

**Table 9:** Comparison of research results.

Research results of this paper	Past research results
Choosing per capita variable can avoid the influence of population based on prediction results to the greatest extent and improve the accuracy of cold chain logistics demand prediction of aquatic products to the greatest extent.	The prediction accuracy of this paper is higher than that of Yan [31] who did not use per capita variable in logistics demand prediction, but lower than that of the research model in this paper.
The traditional grey model, for the samples with obvious trend fluctuations, produces relatively large errors. The neural network has great advantages in dealing with complex nonlinear systems. Combining the traditional grey model with BP neural network can make a more accurate prediction of cold chain logistics demand.	Li et al. [32] used a single model for comparison, instead of combining multiple models for comparative prediction.
The grey prediction model and BP neural network model are the most effective prediction methods for cold chain logistics of aquatic products, which can accurately analyze the nonlinear relationship and contribute to the sustainable development of the cold chain logistics industry.	Xu et al. [33] proved that by combining GM (1,1) prediction with BP neural network, the grey-BP neural network could effectively deal with the demand prediction of cold chain logistics of aquatic products affected by strong complexity, nonlinearity, and multiple factors, to achieve a high degree of nonlinear relationship fitting. But their study had a small sample of weak predictions.

Based on the analysis presented above, this paper makes some suggestions on the reasons that can cause the decline in the demand for aquatic products in China.

1. As China enters a new stage of development, the demand of people for high-quality consumer goods and the demand of market entities for high-quality logistics services are accelerating. It strives to build new channels for two-way cold chain logistics of consumer goods to better meet people’s growing needs for a better life. Government policies can actively lead the development direction of the cold chain logistics industry under the new pattern, which is the guide to action for development in the new era and can better promote China’s economic development. Japan’s decision to discharge nuclear wastewater into the sea is bound to have a huge impact on China’s fishery, aquatic product consumption structure of residents, cold chain logistics industry, and trade, and cause heavy damage to China’s economy and society. Given, the possible discharge of nuclear sewage from Japan, the Chinese government should take measures to actively respond, regularly, and closely monitor water quality in China’s sea areas, and publicize the data, so that the masses can grasp changes in water quality in time, and at the same time strictly monitor the quality of imported seafood to avoid the masses eating contaminated aquatic products by mistake.
2. With the continuous improvement of the consumption level of residents, the demand for fresh agricultural products, medicine, and other cold chain logistics in China is increasing. In the context of the novel coronavirus epidemic, cold chain logistics has become a key area of concern. The change in the consumption pattern of the Chinese people in the post-COVID-19 era has brought great impetus to the development of the cold chain logistics industry. In the context of the post-epidemic era, the price of aquatic products in China has increased greatly in recent years. The government should closely monitor the price trend of aquatic products and control the market when necessary, to avoid people choosing other substitutes for nutritional supplements because of the high price of aquatic products, and to make consumers’ diet structure change continuously.
3. Faced with increasingly fierce market competition and the increasing requirements of people for aquatic products, mariculture enterprises should take a serious, scientific and serious attitude, actively cooperate with the government to carry out water quality inspections, guide people to pay attention to water quality status, and actively publicize the current water quality status in the marketing process, encourage consumers to reduce their health doubts, and achieve the goal of purchasing and eating aquatic products with peace of mind. At the same time, from the consumer’s point of view, we constantly innovate, constantly improve our cold chain logistics level, and reduce logistics costs from the aspects of equipment, technology, quality, and service. The cold chain logistics industry should strictly implement the support policies formulated by the state, study and formulate support policies suitable for the development of the cold chain logistics industry such as land, transportation, and taxation, and resolutely implement the "green channel" policy for the transportation of fresh agricultural products to ensure the stable price of aquatic products.
